# Legume Allergens Pea, Chickpea, Lentil, Lupine and Beyond

**DOI:** 10.1007/s11882-024-01165-7

**Published:** 2024-07-11

**Authors:** Marua Abu Risha, Eva-Maria Rick, Melanie Plum, Uta Jappe

**Affiliations:** 1 Clinical and Molecular Allergology, Priority Research Area Chronic Lung Diseases, Research Center Borstel, Borstel, Germany; 2 German Center for Lung Research (DZL), Airway Research Center North (ARCN), Borstel, Germany; 3 Interdisciplinary Allergy Outpatient Clinic, Department of Pneumology, University of Lübeck, Lübeck, Germany

**Keywords:** Allergen characterization, Anaphylaxis, BAT, FDEIA, Peanut, Soy

## Abstract

**Purpose of the Review:**

In the last decade, an increasing trend towards a supposedly healthier vegan diet could be observed. However, recently, more cases of allergic reactions to plants and plant-based products such as meat-substitution products, which are often prepared with legumes, were reported. Here, we provide the current knowledge on legume allergen sources and the respective single allergens. We answer the question of which legumes beside the well-known food allergen sources peanut and soybean should be considered for diagnostic and therapeutic measures.

**Recent Findings:**

These “non-priority” legumes, including beans, pea, lentils, chickpea, lupine, cowpea, pigeon pea, and fenugreek, are potentially new important allergen sources, causing mild-to-severe allergic reactions. Severe reactions have been described particularly for peas and lupine. An interesting aspect is the connection between anaphylactic reactions and exercise (food-dependent exercise-induced anaphylaxis), which has only recently been highlighted for legumes such as soybean, lentils and chickpea. Most allergic reactions derive from IgE cross-reactions to homologous proteins, for example between peanut and lupine, which is of particular importance for peanut-allergic individuals ignorant to these cross-reactions.

**Summary:**

From our findings we conclude that there is a need for large-scale studies that are geographically distinctive because most studies are case reports, and geographic differences of allergic diseases towards these legumes have already been discovered for well-known “Big 9” allergen sources such as peanut and soybean. Furthermore, the review illustrates the need for a better molecular diagnostic for these emerging non-priority allergen sources to evaluate IgE cross-reactivities to known allergens and identify true allergic reactions.

## Introduction

**Fig. 1:**
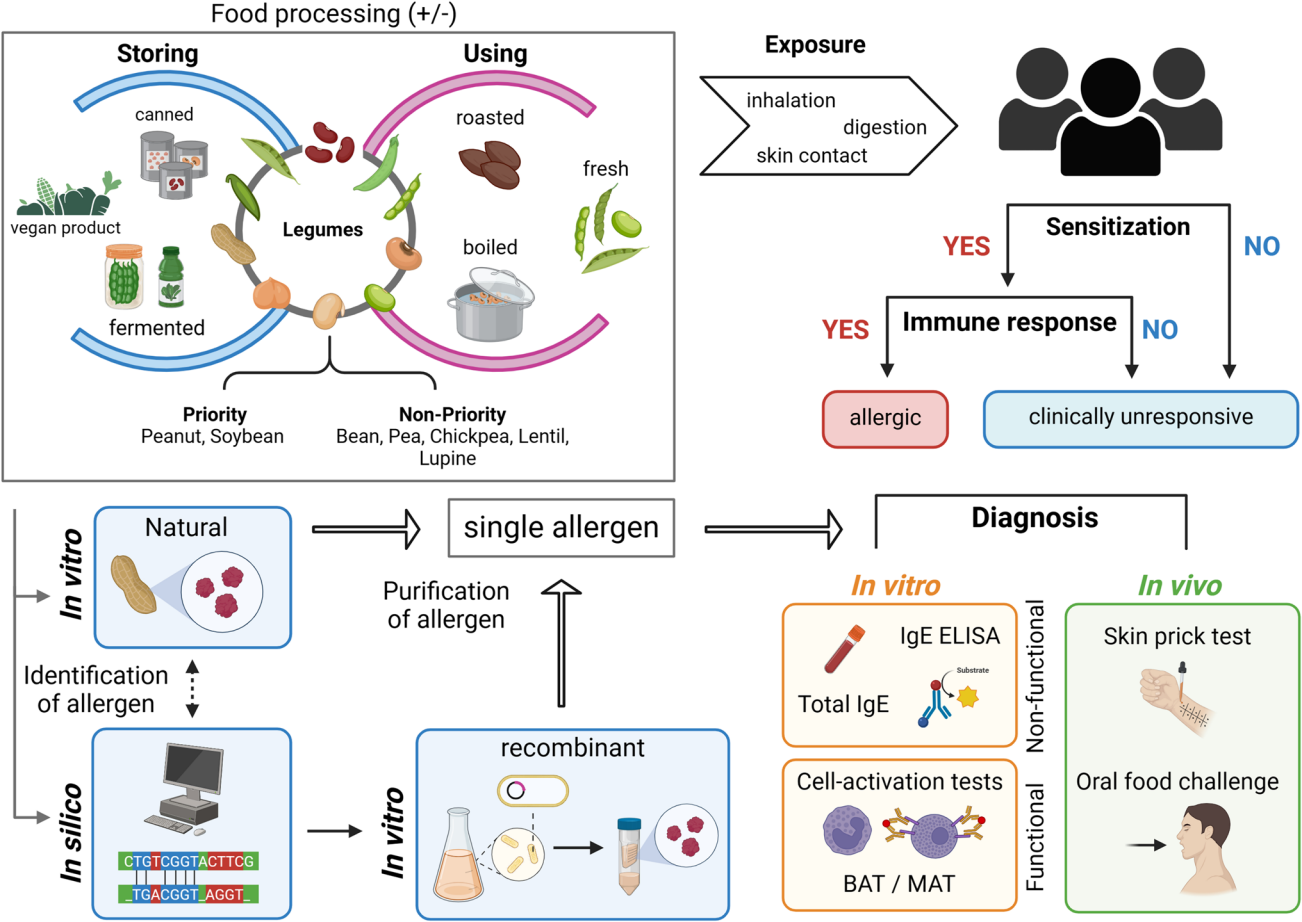
Legume allergy investigation. Identification of single allergens mainly occurs via protein extraction from whole extracts using sera from well-characterized allergic patients in order to show their IgE-binding capacity in vitro. In addition, the in silico approach can be used. After identification of allergens in a whole extract they are purified from the natural source or produced recombinantly. The purified or recombinant allergen can subsequently be applied in diagnostic tests such as the basophil activation test (BAT) and mast cell activation test (MAT).

Within the last decade, the demand for plant-based dietary products including legumes has increased drastically, fueled by the rising trend of the vegan diet which is perceived as healthier and more environmentally-friendly [1]. This prompts a crucial question: Is this diet sufficient and devoid of any adverse effects on human health? The family of *Leguminosae* (legumes) is abundant in proteins, essential nutritional compounds and minerals [2]. In addition, their fiber components were classified as resistant starch that can be broken down by gut bacteria into short-chain fatty acids (SCFAs) which play a crucial role in energy production [3] and also seem to be involved in the maintenance of the skin barrier, reducing susceptibility to allergens [4]. In the last decades, a worldwide rise in cases of allergy was recorded. Reasons for this are complex and maybe related to epigenetic factors influenced by lifestyle, pollution, climate change and other environmental factors. EU regulation 1169/2011 (document 02011R1169-20,180,101) mandates labelling known allergen sources such as soybeans, peanuts and lupines. However, some products may contain unlisted allergens from other sources due to low case numbers of reported allergic reactions. In this review, we focus on the emerging allergens from legumes. But why legumes? Legumes carry allergens of high potency that can trigger intense anaphylactic reactions. These allergens include lipid transfer proteins (LTPs), storage proteins, pathogenesis-related (PR) proteins and structural proteins, which can be resistant to heat and digestive proteases [5, 6]. The change into a more plant-based diet leads to an increased consumption of highly processed plant-based protein products (tofu, soy milk, meat substitute products, etc.) containing concentrated plant proteins, which could trigger allergic symptoms ranging from mild oral allergy syndrome (OAS) to life-threatening anaphylactic reactions [7, 8]. It is crucial to acknowledge that individuals can react differently to allergens depending on the source, consumed status (fresh, boiled/cooked, fried, dried, roasted or fermented) and dietary practices in diverse geographical areas [9, 10]. Different assays and tests are used for allergy diagnostics. The techniques behind them differ depending on the assay (in vivo, in vitro, in silico) (Fig. 1).

Allergen sources from legumes are categorized into two groups: priority and non-priority legumes. The priority group includes the legumes peanuts and soybeans listed within the "Big 9" major food allergens described by the Food and Drug Administration's (FDA). Legumes excluded from the “Big 9” due to low incidence of allergy – the non-priority legume allergen sources – include beans, peas, lentils, chickpeas, lupines, fenugreek, and others. Except for lupine, allergic reactions to these legumes have mostly been described in case reports. As these case reports, however, have accumulated over the past decade or so, this review will put a special emphasis on the non-priority legumes. Details on the legume allergens including tests, molecular weight, allergen source and protein function are presented in Table 1 combining the information from the World Health Organization/International Union of Immunological Societies (WHO/IUIS) Allergen Nomenclature Sub-Committee and the Allergome database. Single legume allergen classifications are listed in Table 2.

**Table 1:** Single allergens or allergen homologs identified from legumes

**Species**	**Known Name**	**Database**	**Allergen or allergen homolog**	**MW** **(kDa)**	**In vivo**	**In vitro** **(functional)**	**In vitro** **(non-functional)**	**Source form**	**Biological function**	**Ref**
*Arachis hypogaea*	**Peanut**	WHO/IUIS	Ara h 1	64	PST	MAT BHRA	IgE & IgE-Immunoblotting & Cross-reactive Carbohydrate Determinant	Natural & Recombinant	7S Vicilin-like Globulin	[11–14]
*Arachis hypogaea*	**Peanut**	WHO/IUIS	Ara h 1.0101	60			IgE-Immunoblotting	Recombinant	7S Vicilin-like Globulin	[11, 15–17]
*Arachis hypogaea*	**Peanut**	WHO/IUIS	Ara h 2	17	PST	BAT MAT BHRA	IgE & IgE-Immunoblotting	Natural & Recombinant	2S Albumin	[13, 14, 18–22]
*Arachis hypogaea*	**Peanut**	WHO/IUIS	Ara h 2.0101	16.57			IgE	Natural & Recombinant	2S Albumin	[18, 19, 23, 24]
*Arachis hypogaea*	**Peanut**	WHO/IUIS	Ara h 2.0201	18			IgE	Recombinant	2S Albumin	[18, 19]
*Arachis hypogaea*	**Peanut**	WHO/IUIS	Ara h 3	60	PST	MAT BHRA	IgE & IgE-Immunoblotting & Cross-reactive Carbohydrate Determinant	Natural & Recombinant	Legumin-like Protein	[12, 13, 18, 25–28]
*Arachis hypogaea*	**Peanut**	WHO/IUIS	Ara h 3.0101				IgE-Immunoblotting	Recombinant	Legumin-like Protein	[25]
*Arachis hypogaea*	**Peanut**	WHO/IUIS	Ara h 3.0201	58.84			IgE-Immunoblotting	Recombinant	Legumin-like Protein	[18]
*Arachis hypogaea*	**Peanut**	WHO/IUIS	Ara h 5	15			IgE & IgE-Immunoblotting	Natural & Recombinant	Profilin	[18, 29–31]
*Arachis hypogaea*	**Peanut**	WHO/IUIS	Ara h 5.0101	14			IgE-Immunoblotting	Recombinant	Profilin	[18]
*Arachis hypogaea*	**Peanut**	WHO/IUIS	Ara h 6	15	PST	BAT BHRA	IgE & IgE-Immunoblotting	Natural & Recombinant	2S Albumin	[18, 22]
*Arachis hypogaea*	**Peanut**	WHO/IUIS	Ara h 6.0101	14.5		BAT	IgE & IgE-Immunoblotting	Recombinant	2S Albumin	[18]
*Arachis hypogaea*	**Peanut**	WHO/IUIS	Ara h 7	15		BAT	IgE & IgE-Immunoblotting	Natural & Recombinant	2S Albumin	[18, 32, 33]
*Arachis hypogaea*	**Peanut**	WHO/IUIS	Ara h 7.0101	< 20			IgE-Immunoblotting	Natural & Recombinant	2S Albumin	[32]
*Arachis hypogaea*	**Peanut**	WHO/IUIS	Ara h 7.0201	17.34		BAT	IgE-Immunoblotting	Natural & Recombinant	2S Albumin	[32, 33]
*Arachis hypogaea*	**Peanut**	WHO/IUIS	Ara h 7.0301	15.7			IgE-Immunoblotting	Natural	2S Albumin	[32]
*Arachis hypogaea*	**Peanut**	WHO/IUIS	Ara h 8	17		BHRA	IgE & IgE-Immunoblotting	Recombinant	PR-Protein	[34–36]
*Arachis hypogaea*	**Peanut**	WHO/IUIS	Ara h 8.0101	17			IgE-Immunoblotting	Recombinant	PR-Protein	[34]
*Arachis hypogaea*	**Peanut**	WHO/IUIS	Ara h 8.0201	16.28			IgE-Immunoblotting	Recombinant	PR-Protein	[36]
*Arachis hypogaea*	**Peanut**	WHO/IUIS	Ara h 9	9.8		BAT BHRA	IgE & IgE-Immunoblotting	Natural & Recombinant	nsLTP	[37–39]
*Arachis hypogaea*	**Peanut**	WHO/IUIS	Ara h 9.0101	9.13		BAT	IgE & IgE-Immunoblotting	Natural & Recombinant	nsLTP	[37, 38]
*Arachis hypogaea*	**Peanut**	WHO/IUIS	Ara h 9.0201	9.04		BAT	IgE & IgE-Immunoblotting	Natural & Recombinant	nsLTP	[37, 38]
*Arachis hypogaea*	**Peanut**	WHO/IUIS	Ara h 10	16		BAT/MAT	IgE & IgE-Immunoblotting	Natural & Recombinant	Oleosin	[40–42]
*Arachis hypogaea*	**Peanut**	WHO/IUIS	Ara h 10.0101	16			IgE & IgE-Immunoblotting	Natural	Oleosin	[40, 41]
*Arachis hypogaea*	**Peanut**	WHO/IUIS	Ara h 10.0102	16			IgE & IgE-Immunoblotting	Natural & Recombinant	Oleosin	[40–42]
*Arachis hypogaea*	**Peanut**	WHO/IUIS	Ara h 11	14		BAT	IgE & IgE-Immunoblotting	Natural & Recombinant	Oleosin	[40, 41]
*Arachis hypogaea*	**Peanut**	WHO/IUIS	Ara h 11.0101	14			IgE & IgE-Immunoblotting	Natural & Recombinant	Oleosin	[40, 42]
*Arachis hypogaea*	**Peanut**	WHO/IUIS	Ara h 11.0102	14.35			IgE & IgE-Immunoblotting	Natural	Oleosin	[40]
*Arachis hypogaea*	**Peanut**	WHO/IUIS	Ara h 12	< 12		BAT	IgE-Immunoblotting	Natural	Defensin	[43]
*Arachis hypogaea*	**Peanut**	WHO/IUIS	Ara h 12.0101	5.19		BAT	IgE-Immunoblotting	Natural	Defensin	[43]
*Arachis hypogaea*	**Peanut**	WHO/IUIS	Ara h 13	< 11		BAT	IgE-Immunoblotting	Natural	Defensin	[43]
*Arachis hypogaea*	**Peanut**	WHO/IUIS	Ara h 13.0101	5.48		BAT	IgE-Immunoblotting	Natural	Defensin	[43]
*Arachis hypogaea*	**Peanut**	WHO/IUIS	Ara h 13.0102	< 8		BAT	IgE-Immunoblotting	Natural	Defensin	[43]
*Arachis hypogaea*	**Peanut**	WHO/IUIS	Ara h 14	17.5		BAT	IgE	Natural	Oleosin	[40, 41]
*Arachis hypogaea*	**Peanut**	WHO/IUIS	Ara h 14.0101	18.435		BAT	IgE	Natural	Oleosin	[40, 44]
*Arachis hypogaea*	**Peanut**	WHO/IUIS	Ara h 14.0102	18.457		BAT	IgE	Natural	Oleosin	[40, 44]
*Arachis hypogaea*	**Peanut**	WHO/IUIS	Ara h 14.0103	18.448		BAT	IgE	Natural	Oleosin	[40]
*Arachis hypogaea*	**Peanut**	WHO/IUIS	Ara h 15	17		BAT	IgE & IgE-Immunoblotting	Natural & Recombinant	Oleosin	[40–42, 45]
*Arachis hypogaea*	**Peanut**	WHO/IUIS	Ara h 15.0101	16.87		BAT	IgE	Natural	Oleosin	[40]
*Arachis hypogaea*	**Peanut**	WHO/IUIS	Ara h 16	8.5			IgE-Immunoblotting	Natural	nsLTP	[46]
*Arachis hypogaea*	**Peanut**	WHO/IUIS	Ara h 16.0101	7.01			IgE-Immunoblotting	Natural	nsLTP	[46]
*Arachis hypogaea*	**Peanut**	WHO/IUIS	Ara h 17	11			IgE-Immunoblotting	Natural	nsLTP	[46]
*Arachis hypogaea*	**Peanut**	WHO/IUIS	Ara h 17.0101	9.3			IgE-Immunoblotting	Natural	nsLTP	[46]
*Arachis hypogaea*	**Peanut**	WHO/IUIS	Ara h 18	21				Natural	Cyclophilin—peptidyl-prolyl cis–trans isomerase	[47]
*Arachis hypogaea*	**Peanut**	WHO/IUIS	Ara h 18.0101	18.2				Natural	Cyclophilin—peptidyl-prolyl cis–trans isomerase	[47]
*Arachis hypogaea*	**Peanut**	Allergome	Ara h Agglutinin	29			IgE & IgE-Immunoblotting	Natural	Agglutinin	[48–51]
*Cajanus cajan*	**Red gram (Pigeon peas)**	Allergome	Caj ca 1	45			IgE-Immunoblotting	Natural	Vicilin-like Protein	[52]
*Cicer arietinum*	**Chickpea**	WHO/IUIS	Cic a 1	42			IgE-Immunoblotting	Natural	Late embryogenesis protein 4	[53, 54]
*Cicer arietinum*	**Chickpea**	WHO/IUIS	Cic a 1.0101	34.6			IgE-Immunoblotting	Recombinant	Late embryogenesis protein 4	[53, 54]
*Cicer arietinum*	**Chickpea**	Allergome	Cic a 2S Albumin	20			IgE & IgE-Immunoblotting	Natural	2S Albumin	[55]
*Cicer arietinum*	**Chickpea**	Allergome	Cic a 3					In silico	nsLTP	[56]
*Cicer arietinum*	**Chickpea**	Allergome	Cic a 4					In silico	PR-Protein	[56]
*Cicer arietinum*	**Chickpea**	Allergome	Cic a 6	56			IgE-Immunoblotting	Natural	Legumin-like Protein	[53]
*Cicer arietinum*	**Chickpea**	Allergome	Cic a 10	70			IgE-Immunoblotting	Natural	Luminal-binding Protein	[53]
*Cicer arietinum*	**Chickpea**	Allergome	Cic a Albumin	26			IgE	Natural	Albumin	[57, 58]
*Cyamopsis tetragonoloba*	**Guar bean (cluster bean)**	Allergome	no allergen has been identified yet							[59–61]
*Glycine max*	**Soybean**	WHO/IUIS	Gly m 1	7			IgE & IgE-Immunoblotting	Natural	Hydrophobic Seed Protein	[62]
*Glycine max*	**Soybean**	WHO/IUIS	Gly m 1.0101	8			IgE-Immunoblotting	Natural	Hydrophobic Seed Protein	[63]
*Glycine max*	**Soybean**	WHO/IUIS	Gly m 1.0102	8			IgE-Immunoblotting	Natural	Hydrophobic Seed Protein	[63]
*Glycine max*	**Soybean**	WHO/IUIS	Gly m 2	8			IgE & IgE-Immunoblotting	Natural	Defensin	[64–66]
*Glycine max*	**Soybean**	WHO/IUIS	Gly m 2.0101	8			IgE	Natural	Defensin	[64]
*Glycine max*	**Soybean**	WHO/IUIS	Gly m 3	14			IgE & IgE-Immunoblotting	Natural	Profilin	[67, 68]
*Glycine max*	**Soybean**	WHO/IUIS	Gly m 3.0101	14.1			IgE & IgE-Immunoblotting	Recombinant	Profilin	[67]
*Glycine max*	**Soybean**	WHO/IUIS	Gly m 3.0102	14.1			IgE & IgE-Immunoblotting	Recombinant	Profilin	[67]
*Glycine max*	**Soybean**	WHO/IUIS	Gly m 4	17		BAT HR	IgE & IgE-Immunoblotting	Natural	PR-Protein	[69–73]
*Glycine max*	**Soybean**	WHO/IUIS	Gly m 4.0101	17		BAT HR	IgE & IgE-Immunoblotting	Recombinant	PR-Protein	[69, 73, 74]
*Glycine max*	**Soybean**	WHO/IUIS	Gly m 5	48		BAT	IgE & IgE-Immunoblotting	Natural	Vicilin-like Globulin	[73, 75, 76]
*Glycine max*	**Soybean**	WHO/IUIS	Gly m 5.0101	63.16		BAT	IgE & IgE-Immunoblotting	Natural	Vicilin-like Globulin	[73, 77–81]
*Glycine max*	**Soybean**	WHO/IUIS	Gly m 5.0201	65.14		BAT	IgE & IgE-Immunoblotting	Natural	Vicilin-like Globulin	[73, 77–79]
*Glycine max*	**Soybean**	WHO/IUIS	Gly m 5.0301	47.9		BAT	IgE & IgE-Immunoblotting	Natural	Vicilin-like Globulin	[73, 77–79]
*Glycine max*	**Soybean**	WHO/IUIS	Gly m 5.0302	47.97		BAT	IgE & IgE-Immunoblotting	Natural	Vicilin-like Globulin	[73, 77–79]
*Glycine max*	**Soybean**	WHO/IUIS	Gly m 6	55		BAT	IgE	Natural	Legumin-like Protein	[73, 77, 82]
*Glycine max*	**Soybean**	WHO/IUIS	Gly m 6.0101	53.62			IgE & IgE-Immunoblotting	Recombinant	Legumin-like Protein	[83–85]
*Glycine max*	**Soybean**	WHO/IUIS	Gly m 6.0201	52.44			IgE & IgE-Immunoblotting	Recombinant	Legumin-like Protein	[86, 87]
*Glycine max*	**Soybean**	WHO/IUIS	Gly m 6.0301	52.19			IgE & IgE-Immunoblotting	Recombinant	Legumin-like Protein	[77]
*Glycine max*	**Soybean**	WHO/IUIS	Gly m 6.0401	61.41			IgE & IgE-Immunoblotting	Recombinant	Legumin-like Protein	[88]
*Glycine max*	**Soybean**	WHO/IUIS	Gly m 6.0501	55.65			IgE & IgE-Immunoblotting	Recombinant	Legumin-like Protein	[77]
*Glycine max*	**Soybean**	WHO/IUIS	Gly m 7	76.2		BAT	IgE & IgE-Immunoblotting	Natural	Seed Biotinylated Protein	[89]
*Glycine max*	**Soybean**	WHO/IUIS	Gly m 7.0101	67.95		BAT	IgE & IgE-Immunoblotting	Recombinant	Seed Biotinylated Protein	[89]
*Glycine max*	**Soybean**	WHO/IUIS	Gly m 8	28			IgE	Natural	2S Albumin	[90–93]
*Glycine max*	**Soybean**	WHO/IUIS	Gly m 8.0101	28			IgE & IgE-Immunoblotting	Recombinant	2S Albumin	[90–93]
*Glycine max*	**Soybean**	Allergome	Gly m Agglutinin	30.9			IgE & IgE-Immunoblotting	Natural	Agglutinin	[50]
*Glycine max*	**Soybean**	Allergome	Gly m Bd28K	28			IgE & IgE-Immunoblotting & Cross-reactive Carbohydrate Determinant	Natural & Recombinant	7S Vicilin-like Globulin	[94]
*Glycine max*	**Soybean**	Allergome	Gly m Bd30K	32–34			IgE & IgE-Immunoblotting	Natural & Recombinant	Protease	[95, 96]
*Glycine max*	**Soybean**	Allergome	Gly m 39kD	39			IgE & IgE-Immunoblotting	Natural & Recombinant	Unknown	[92, 97]
*Glycine max*	**Soybean**	Allergome	Gly m 50kD	50			IgE-Immunoblotting	Natural	Unknown	[98]
*Glycine max*	**Soybean**	Allergome	Gly m CPI	25			IgE-Immunoblotting	Natural	Cysteine Protease Inhibitor	[99]
*Glycine max*	**Soybean**	Allergome	Gly m EAP	60			IgE-Immunoblotting	Natural	Embryonic Abundant Protein	[99]
*Glycine max*	**Soybean**	Allergome	Gly m TI	21.5	PST		IgE & IgE-Immunoblotting	Natural	Trypsin Inhibitor	[48]
*Lablab purpureus*	**Hyacinth bean (Lablab)**	Allergome	no allergen has been identified yet							[100]
*Lathyrus sativus*	**grass pea (white pea)**	Allergome	no allergen has been identified yet							[101–105]
*Lens culinaris*	**Lentil**	WHO/IUIS	Len c 1	44–47				Natural & Recombinant	7S Vicilin-like Globulin	[106–108]
*Lens culinaris*	**Lentil**	WHO/IUIS	Len c 1.0101	44–47				Recombinant	7S Vicilin-like Globulin	[107]
*Lens culinaris*	**Lentil**	WHO/IUIS	Len c 1.0102	44–47				Recombinant	7S Vicilin-like Globulin	[107, 109]
*Lens culinaris*	**Lentil**	WHO/IUIS	Len c 1.0103						7S Vicilin-like Globulin	[107]
*Lens culinaris*	**Lentil**	WHO/IUIS	Len c 2	66			IgE & IgE-Immunoblotting	Natural	Seed Biotinylated Protein	[106]
*Lens culinaris*	**Lentil**	WHO/IUIS	Len c 2.0101						Seed Biotinylated Protein	[106]
*Lens culinaris*	**Lentil**	WHO/IUIS	Len c 3	9			IgE	Natural	nsLTP	[110, 111]
*Lens culinaris*	**Lentil**	WHO/IUIS	Len c 3.0101	9,283			IgE	Recombinant	nsLTP	[111]
*Lens culinaris*	**Lentil**	Allergome	Len c Agglutinin	30.35			IgE	Natural	Agglutinin	[50]
*Lupinus albus*	**White Lupine**	Allergome	Lup a 1	62	PST		IgE-Immunoblotting	Natural	7S Vicilin-like Globulin	[112–114]
*Lupinus albus*	**White Lupine**	Allergome	Lup a 4					In silico	PR-Protein	[115, 116]
*Lupinus albus*	**White Lupine**	WHO/IUIS	Lup a 5	15			IgE-Immunoblotting	Recombinant	Profilin	[117]
*Lupinus albus*	**White Lupine**	WHO/IUIS	Lup a 5.0101	13.9			IgE-Immunoblotting	Recombinant	Profilin	[117]
*Lupinus albus*	**White Lupine**	Allergome	Lup a alpha_Conglutin	20	PST		IgE-Immunoblotting	Natural	Legumin-like Protein	[112, 113, 118]
*Lupinus albus*	**White Lupine**	Allergome	Lup a delta_Conglutin	17	PST			Natural	2S Albumin	[112, 119]
*Lupinus albus*	**White Lupine**	Allergome	Lup a gamma_Conglutin	62.1	PST		IgE-Immunoblotting	Natural	Aspartic Protease	[112, 113, 120, 121]
*Lupinus angustifolius*	**Blue Lupine**	WHO/IUIS	Lup an 1	55–61			IgE & IgE-Immunoblotting	Natural	7S Vicilin-like Globulin	[122–124]
*Lupinus angustifolius*	**Blue Lupine**	WHO/IUIS	Lup an 1.0101	55–61			IgE	Natural	7S Vicilin-like Globulin	[125]
*Lupinus angustifolius*	**Blue Lupine**	WHO/IUIS	Lup an 3	11			IgE & IgE-Immunoblotting	Natural	nsLTP	[117]
*Lupinus angustifolius*	**Blue Lupine**	WHO/IUIS	Lup an 3.0101	9.23			IgE & IgE-Immunoblotting	Natural	nsLTP	[117, 126]
*Lupinus angustifolius*	**Blue Lupine**	Allergome	Lup an alpha_Conglutin	15.53			IgE & IgE-Immunoblotting	Natural	Legumin-like Protein	[113, 124]
*Lupinus angustifolius*	**Blue Lupine**	Allergome	Lup an delta_Conglutin	17.78			IgE & IgE-Immunoblotting	Natural	2S Albumin	[124, 127]
*Lupinus angustifolius*	**Blue Lupine**	Allergome	Lup an gamma_Conglutin	48.91			IgE & IgE-Immunoblotting	Natural	Aspartic Protease	[113, 124, 127, 128]
*Lupinus luteus*	**Yellow Lupine (European Lupine)**	Allergome	Lup l 4	16.859			IgE	Recombinant	PR-Protein	[129, 130]
*Macrotyloma uniflorum*	**Horse gram (kulthi bean)**	Allergome	Dol b Agglutinin	26	PST	HR	IgE & IgE-Immunoblotting	Natural	Agglutinin	[131]
*Pachyrhizus erosus*	**Yam-bean**	Allergome	no allergen has been identified yet							[132, 133]
*Phaseolus coccineus*	**Runner Bean**	Allergome	no allergen has been identified yet							[134]
*Phaseolus lunatus*	**Lima bean**	Allergome	no allergen has been identified yet							[135, 136]
*Phaseolus vulgaris*	**Kidney Bean (String Bean)**	WHO/IUIS	Pha v 3	9		HR	IgE & IgE-Immunoblotting	Natural & Recombinant	nsLTP	[137–139]
*Phaseolus vulgaris*	**Kidney Bean (String Bean)**	WHO/IUIS	Pha v 3.0101	9.375		HR	IgE-Immunoblotting	Recombinant	nsLTP	[137–139]
*Phaseolus vulgaris*	**Kidney Bean (String Bean)**	WHO/IUIS	Pha v 3.0201	9.221		HR	IgE-Immunoblotting	Recombinant	nsLTP	[137–139]
*Phaseolus vulgaris*	**Kidney Bean (String Bean)**	Allergome	Pha v 5					In silico	Profilin	[140]
*Phaseolus vulgaris*	**Kidney Bean (String Bean)**	Allergome	Pha v 6					In silico	PR-Protein	
*Phaseolus vulgaris*	**Kidney Bean (String Bean)**	Allergome	Pha v aAI					Recombinant	alpha-Amylase Inhibitor	[141]
*Phaseolus vulgaris*	**Kidney Bean (String Bean)**	Allergome	Pha v aAI.0101	27.2				Recombinant	alpha-Amylase Inhibitor	[141]
*Phaseolus vulgaris*	**Kidney Bean (String Bean)**	Allergome	Pha v Chitinase	32			IgE-Immunoblotting	Natural	Chitinase	[142]
*Phaseolus vulgaris*	**Kidney Bean (String Bean)**	Allergome	Pha v PHA	29.55	PST	BHRA	IgE & IgE-Immunoblotting	Natural	Agglutinin	[143, 144]
*Phaseolus vulgaris*	**Kidney Bean (String Bean)**	Allergome	Pha v Phaseolin	47.5	PST		IgE-Immunoblotting	Natural	Vicilin-like Protein	[145, 146]
*Pisum sativum*	**Pea**	WHO/IUIS	Pis s 1	44–47			IgE & IgE-Immunoblotting	Natural & Recombinant	Vicilin-like Protein	[108, 147]
*Pisum sativum*	**Pea**	WHO/IUIS	Pis s 1.0101	44			IgE & IgE-Immunoblotting	Recombinant	Vicilin-like Protein	[108]
*Pisum sativum*	**Pea**	WHO/IUIS	Pis s 1.0102	44			IgE & IgE-Immunoblotting	Recombinant	Vicilin-like Protein	[108]
*Pisum sativum*	**Pea**	WHO/IUIS	Pis s 2	63			IgE-Immunoblotting	Natural	7S Vicilin-like Globulin	[108, 148]
*Pisum sativum*	**Pea**	WHO/IUIS	Pis s 2.0101	67			IgE	Recombinant	7S Vicilin-like Globulin	[108]
*Pisum sativum*	**Pea**	WHO/IUIS	Pis s 3	9.5			IgE	Natural	nsLTP	[149]
*Pisum sativum*	**Pea**	WHO/IUIS	Pis s 3.0101	9.5			IgE	Recombinant	nsLTP	[149]
*Pisum sativum*	**Pea**	Allergome	Pis s 5					Natural	Profilin	[29, 150]
*Pisum sativum*	**Pea**	Allergome	Pis s 6					In silico	PR-Protein	[151]
*Pisum sativum*	**Pea**	Allergome	Pis s Agglutinin	30.27			IgE	Natural	Agglutinin	[50]
*Pisum sativum*	**Pea**	Allergome	Pis s Albumin	26.23			IgE	Natural	Albumin	[57, 148]
*Trigonella foenum-graecum*	**Fenugreek**	Allergome	Tri fg 1	50			IgE-Immunoblotting	Natural	7S Vicilin-like Globulin	[152]
*Trigonella foenum-graecum*	**Fenugreek**	Allergome	Tri fg 2	98			IgE-Immunoblotting	Natural	2S Albumin	[152]
*Trigonella foenum-graecum*	**Fenugreek**	Allergome	Tri fg 3	98			IgE-Immunoblotting	Natural	Legumin-like Protein	[152]
*Trigonella foenum-graecum*	**Fenugreek**	Allergome	Tri fg 4	21			IgE-Immunoblotting	Natural	PR-Protein	[152]
*Vicia faba*	**Broad Bean**	Allergome	Vic f 6					In silico	PR-Protein	
*Vigna angularis*	**Red Mung bean (Azuki Bean)**	Allergome	Vig an 6					In silico	PR-Protein	
*Vigna mungo*	**Black gram**	Allergome	Vig mu 28kD	28	PST	LPA & BHRA	IgE & IgE-Immunoblotting	Natural	Unknown	[153]
*Vigna radiata*	**Green gram** **(Mung bean)**	WHO/IUIS	Vig r 1	16		BAT	IgE-Immunoblotting	Natural & Recombinant	PR-Protein	[74, 154, 155]
*Vigna radiata*	**Green gram** **(Mung bean)**	WHO/IUIS	Vig r 1.0101	16.2		BAT	IgE	Natural & Recombinant	PR-Protein	[74, 154, 155]
*Vigna radiata*	**Green gram** **(Mung bean)**	WHO/IUIS	Vig r 2	52			IgE-Immunoblotting	Natural	8S Globulin	[156]
*Vigna radiata*	**Green gram (Mung bean)**	WHO/IUIS	Vig r 2.0101	49.3			IgE-Immunoblotting	Recombinant	8S Globulin	[156, 157]
*Vigna radiata*	**Green gram (Mung bean)**	WHO/IUIS	Vig r 2.0201	49.4			IgE-Immunoblotting	Natural	8S Globulin	[156, 158]
*Vigna radiata*	**Green gram (Mung bean)**	WHO/IUIS	Vig r 4	30			IgE-Immunoblotting	Natural	Seed albumin	[74, 156]
*Vigna radiata*	**Green gram (Mung bean)**	WHO/IUIS	Vig r 4.0101	30.2			IgE-Immunoblotting	Natural	Seed albumin	[74, 156]
*Vigna radiata*	**Green gram (Mung bean)**	Allergome	Vig r 5	15			IgE-Immunoblotting	Natural	profilin-homolog	
*Vigna radiata*	**Green gram (Mung bean)**	WHO/IUIS	Vig r 6	18		BAT	IgE	Recombinant	PR-Protein	[74, 129]
*Vigna radiata*	**Green gram (Mung bean)**	WHO/IUIS	Vig r 6.0101	17.49		BAT	IgE	Recombinant	PR-Protein	[74, 129, 159]
*Vigna radiata*	**Green gram (Mung bean)**	Allergome	Vig r beta-Conglycinin	18			IgE-Immunoblotting	Natural	Vicilin-like Protein	[156]
*Vigna unguiculata*	**Cowpea**	Allergome	Vig u 6					In silico	PR-Protein	[160]

The table is arranged in alphabetical order of the Latin names of the allergen source species. Basophil activation test (BAT); mast cell activation test (MAT); stripped basophil histamine release assay (BHRA); histamine release assay (HR); lymphoproliferation assay (LPA); non-specific lipid transfer proteins (nsLTP). Allergens sourced from Allergome are not recognized as official. Only those listed in the WHO/IUIS Allergen Nomenclature Sub-Committee database hold official status.

**Table 2:** Classification of single legume allergen and allergen homologs by protein families

**Source/Function**	**7S globulins** **(vicilins)**	**11S globulins (legumins)**	**2S albumins**	**Profilins**	**PR proteins**	**Biotinylated Proteins**	**nsLTP**	**Agglutinins**	**Defensins**	**Oleosins**	**Diverse**
**Peanut**	Ara h 1	Ara h 3	Ara h 2 Ara h 6 Ara h 7	Ara h 5	Ara h 8		Ara h 9 Ara h 16 Ara h 17	Ara h Agglutinin	Ara h 12 Ara h 13	Ara h 10 Ara h 11 Ara h 14 Ara h 15	Ara h 18
**Soybean**	Gly m 5 Gly m Bd28K	Gly m 6	Gly m 8	Gly m 3	Gly m 4	Gly m 7		Gly m Agglutinin	Gly m 2		Gly m 1 Gly m TI Gly m Bd30K Gly m 39kD Gly m 50kD Gly m CPI Gly m EAP
**White Lupine**	Lup a 1	Lup a alpha-Conglutin	Lup a delta-Conglutin	Lup a 5	Lup a 4						
**Blue Lupine**	Lup an 1	Lup an alpha-Conglutin	Lup an delta-Conglutin				Lup an 3				
**Yellow Lupine**					Lup l 4						
**Pea**	Pis s 1 Pis s 2			Pis s 5	Pis s 6		Pis s 3	Pis s Agglutinin			Pis s Albumin
**Lentil**	Len c 1					Len c 2	Len c 3	Len c Agglutinin			
**Chickpea**		Cic a 6	Cic a 2S Albumin		Cic a 4		Cic a 3				Cic a 1 Cic a 10
**Fenugreek**	Tri fg 1	Tri fg 3	Tri fg 2		Tri fg 4						
**Kidney Bean**				Pha v 5	Pha v 6		Pha v 3	Pha v PHA			Pha v aAI Pha v chitinase
**Mung Bean**	Vig r beta-Conglycinin			Vig r 5	Vig r 1 Vig r 6						
**Broad Bean**					Vic f 6						
**Azuki Bean**					Vig an 6						
**Cowpea**					Vig u 6						
**Pigeon Pea**	Caj ca 1										
**Horse Gram**								Dol b Agglutinin			

Non-specific lipid transfer proteins (nsLTP). Allergens sourced from Allergome are not recognized as official. Only those listed in the WHO/IUIS Allergen Nomenclature Sub-Committee database hold official status.

## Priority Legumes

Among the priority legumes, peanuts and soybeans are the most common triggers of anaphylaxis in countries such as Germany, Austria, and Switzerland [161]. Interestingly, these oilseeds [162] can provoke intense allergic reactions, while non-oilseed legumes may cause less obvious responses (hidden allergic reactions). Oil bodies (oleosomes) as part of the lipid content of the seed are stabilized by different proteins (oleosins, steroleosins, caleosins) [40, 163, 164]. Moreover, Schwager et al. showed by basophil activation test (BAT) measurements that oleosins can be used as marker allergens to reliably distinguish peanut allergic patients with severe allergic reactions from simply sensitized individuals [41]. Extracts for diagnostic and therapeutic purposes are aqueous extracts and therefore lack lipophilic compounds such as oleosins. Hidden allergic reactions highlight the importance of identifying allergens in each type of legume to avoid risks when using these plants in the manufactured products consumed worldwide [7, 165] and to improve the quality of diagnostic and therapeutic applications. Peanut (*Arachis hypogaea*) seeds are the most prevalent among all legumes [166].

Peanut allergy symptoms can vary from mild to severe to even life-threatening anaphylactic reactions [167, 168]. Identifying peanut allergens was done extensively in the last decade [40, 41, 43, 47]. Many isoforms from peanuts were characterized over the years (Table 1). People with peanut allergy might also have allergic reactions to other legumes (cross-reactivity): A cohort study with 195 peanut-allergic children showed positive IgE reactivities towards lupine, fenugreek, soy and lentils [169]. However, the clinical relevance of cross-reactivities to soy are questionable [170, 171]. Soybean (*Glycine max*) is also listed in the “Big 9”, however, the recent ad hoc Joint FAO/WHO expert consultation on risk assessment of food allergens [172] recommended to remove soybean from this list as the overall prevalence is rather low and soybean allergy is only predominant in certain geographic areas (2% in Japan [173], 0.6% in the US [174, 175] and 0.5% in Europe [176]) according to self-reported surveys. Eight official soybean allergens are listed in the WHO/IUIS Allergen Nomenclature Sub-Committee database. Eight additional IgE-binding proteins from soybean are also listed in the Allergome database (Table 1). Anaphylactic reactions towards soy are severe for around one third of the cases and are more frequent in adults when compared to children [161]. They are mostly associated with Gly m 3 to 6 [77, 177, 178], and some case studies even suggest exercise as a co-factor of anaphylaxis to soybean allergens (food-dependent exercise-induced anaphylaxis (FDEIA)) [179, 180]. Although sensitization towards profilin (Gly m 3) and PR10 protein (Gly m 4) was the most frequent cause for systemic reactions in a large retrospective study [178], Gly m 4 seems to be the predominating trigger [70, 181]. While it is well-known that the storage proteins Gly m 5 to 8 are resistant to heat and/or digestion [89, 93, 182–184], Gly m 4 was only recently shown to refold after heating and trigger an IgE response, with or without homologous Bet v 1-related epitopes [185]. Furthermore, this allergen can cross the intestinal barrier, especially if the gastric pH is too high for proper digestion [185]. This might explain why soybean-based drinks can be dangerous for people with birch or alder pollen allergy [73].

## Non-Priority Legumes

### Beans

Allergy towards beans is rare and is frequently accompanied by IgE cross-reactivity to other legumes, though exceptions occur as described below. Although beans have fewer known allergens, cross-reactivities with soybeans and peanuts have been reported [165]. The route of sensitization includes the oral mucosa and the respiratory tract as well as the skin (contact urticaria), although only one case has been reported for the latter: An 84-year-old man experienced skin swelling after skin contact with runner bean plants (*Phaseolus coccineus*) in his garden [160]. Bean allergy highly depends on the genus and species of the bean. For instance, allergic reactions to the white bean (*Phaseolus vulgaris*) were highlighted by recent case reports. Matsui et al. reported an allergic reaction to white beans in a child [186]. IgE-reactivity determined by ImmunoCAP occurred to bean allergens such as phytohemagglutinin, Group 3 LEA, lipoxygenase, and legumin. Another report published in 2023 described two children exhibiting allergic reactions to both white and red beans, yet showed no reactions to other legumes. Western blot analysis using sera of two patients that reacted to extracts from white and red beans identified two IgE-binding proteins in the extracts with molecular weights in the ranges of 47–50 kDa and 28–31 kDa [187]. The mung bean (*Vigna radiata*) is used in meat-substitution products, which are consumed worldwide. Two cases of OAS have been described, one in a teenage Japanese girl, and another involving a male in his twenties [188]. Both cases tested positive for IgE specific to soybean Gly m 4 and birch Bet v 1 [154] (Table 1). Lima beans (*Phaseolus lunatus*) induce positive skin test reactions in patients with other legume allergies but with a low percentage (3.4%) as shown by a survey for asthma and rhinitis patients in Delhi [135], which mostly correlates to cross-reactivity with other legumes [189, 190]. No allergen has been identified from broad bean (*Vicia faba*), despite an instance where a 5-year-old boy (no allergy history) experienced an anaphylaxis after eating a legume-based snack containing fried broad beans. The immunoblotting results with 3 different extracts of broad bean (raw, cooked, and fried) were IgE-positive for the fried bean only, detecting allergens at 37, 21, 17, and 15 kDa [191]. Although considered as a food allergen, a rare case related to occupational asthma from inhaling lima bean vapors was reported in 2012, whereby a new worker in a food factory (a 41-year-old male) developed asthma-like symptoms after 4 months of working on boiling, mincing, and drying large amounts of lima beans [192]. A study conducted in India, which included 815 patients suffering from asthma and allergic rhinitis, revealed that 4% of them were hypersensitive to black gram (*Vigna mungo*) [193]. Subsequently, the single IgE-binding protein Vig mu 28 kD from black gram was characterized [153].

### Peas

The high protein content in peas (*Pisum sativum*) makes them an important nutritional source. Their ability to absorb water has led to their widespread use in the production of meat-substitution products. Case reports for pea allergy have been more frequent as of late including descriptions of severe reactions, which indicate that a switch to a plant-based diet can harbor risks for certain individuals. For example, Abi-Melhem et al. [194] described six cases of pea allergy, five of which were children less than 10 years old and a 15-year-old boy with a history of cashew allergy but tolerance to peanut. The latter immediately developed hives on his face and neck in three incidences after consumption of processed food that contained green peas, such as substitute milk, substitute chicken nuggets and pea-based protein powder. The severe response was accompanied by an elevated IgE level to pea extract. Another report described a 28-year-old woman with asthma who had a severe reaction to falafel containing peas [195]. She had a similar reaction to peas when she was 10 years old and has since been cautiously avoiding peas and peanuts. Her skin prick test (SPT) was positive, and specific IgE to pea was slightly increased. However, other legumes, nuts and sesame showed negative in the SPT. These cases raised a question whether peas are safe for people with sensitization to homologous allergens. Cohort data from Italy demonstrated that pea LTP (Pis s 3) is safe to consume for LTP-allergic patients [196], whilst a German cohort study discovered the seed storage protein Pis s 1 as major immunodominant allergen [197].

### Lentils

Lentils (*Lens culinaris*) are a rich source of protein with antioxidant and anti-inflammatory benefits [198, 199]. The lentils’ unique flavor has led to their widespread use in vegan products [8, 200]. Routes of entry include digesting, inhaling and skin contact. Several unique case studies highlighted the potential allergenic risk of lentils. For example, a 9-year-old boy developed anaphylaxis after consuming lentil soup. Following this incident, the boy removed lentils from his diet. However, during a separate incident involving direct skin contact with lentil soup, he experienced itching, redness, and swelling of the skin [201]. Another example involves a 22-months-old girl who developed an allergic reaction shortly after inhaling the vapors from cooking lentils. A positive specific IgE signal for lentils confirmed the lentil sensitization [202]. Further cases include an 8½-year-old boy who experienced swelling of his eyelids, cough and wheezing after exposure to steam from boiling lentils. Similar reactions were reported after eating lentil soup, chickpeas and peas. His blood tests showed eosinophilia and high total IgE levels, and his diagnosis was confirmed via SPT [203]. A 12-year-old girl without any pollen allergy observed an OAS after lentil and cashew consumption. She developed a reaction after having consumed lentil soup in the Kindergarden. A subsequent investigation revealed that the lentil soup contained peanuts (50 g/L) (Jappe, personal communication). Suspecting an underlying reaction to peanuts as the causative agent, the patient underwent diagnostics confirming an IgE reaction to both, peanut (87.10 kU/L) and lentil (6.08 kU/L) extracts. Specific IgE was positive to Ara h 1 (77.6 kU/L), Ara h 2 (14.8 kU/L), and Ara h 3 (1.7 kU/L), but not towards Ara h 8 and Ara h 9. Oral provocation with 4 spoons of lentil soup without peanuts induced OAS, whereas oral provocation with peanuts led to bronchoconstriction, flush and laryngeal edema at a cumulative dose of ca. 14 mg of peanuts (Jappe, personal communication). More recently, a rare case of FDEIA after eating lentils was published by Alnabulsi et al., where a 16-year-old girl experienced respiratory distress with urticaria-like symptoms upon exercising within an hour after eating lentils. These symptoms recurred again under similar circumstances. Interestingly, her IgE levels for lentils were low [204]. A similar reaction was observed for a 17-year-old boy with four different encounters after eating lentils and exercising [205]. These cases highlight the need for more specific tests and a detailed clinical history regarding activities after consumption (e.g. exercising) when investigating lentil allergy due to the observed cross-reactivities with peanuts, peas, and chickpeas [169, 206, 207].

### Chickpea

Chickpeas (*Cicer arietinum*) are among the most frequently eaten legumes and are used in a variety of popular vegan recipes such as falafel and hummus. Despite the low incidence of chickpea allergy, some old reports back to 2008 have documented cases of FDEIA related to eating chickpea. For example, both, a 16-year-old boy [205] and a 17-year-old girl [208] experienced anaphylaxis after eating chickpeas and performing subsequent exercise. Both showed SPT positive results for chickpea, whilst IgE was only determined for the latter, indicating high IgE titers for chickpea and soybean [208]. These cases point out the potential allergenic risk of chickpeas and the need for further research.

### Lupine

*Lupinus* species are comparable to soy with regard to their nutritional value, which is why they are being used not only as ingredient to foods but as food themselves [209–211]. Lupine seeds or the respective flour are used as nutritional substitute for people with allergies or intolerances to wheat, milk, eggs, and gluten [212]. Lupine allergy has first been described in the USA [212], then in Germany [213] and recently in a Canadian child with peanut allergy [214]. Despite lupine being listed on EU food ingredients since 2006 (Directive 2000/13/EC), individuals unaware of their allergy to lupine via cross-reactivity to peanuts, for example, may still be unknowingly exposed. Due to this individually unclear situation, lupine allergens are still considered as “hidden allergens” [7, 215, 216]. Mainly three lupine species are used for human nutrition: *L. angustifolius*, *L. albus*, and *L. luteus*. A fourth species is used in South America (*L. mutabilis)* [215]. Allergy to lupine occurs as mono-allergy or as a result of cross-reactivity between the allergenic proteins of different legumes, of which peanut is the most relevant culprit [215]. The most common patterns of clinical cross-reactivity among legumes are between peanut, lupine, soy, chickpea and lentil, although this is highly dependent on geography and prevalence of these foods in the diet [217]. Despite the lupine’s potential as an anaphylactic allergen source, only three allergens have been accepted by the WHO/IUIS Allergen Nomenclature Sub-Committee due to variations in protein content, allergen composition and structure across different lupine species [117]: Lup an 1 (a β-conglutin of *L. angustifolius*), Lup an 3, a non-specific LTP of *L. angustifolius*, and Lup a 5, the profilin of *L. albus*. Sequences of the same protein in different species and cultivars can differ, for example the β-conglutin in blue lupine is 60–70% identical to β-conglutin of white lupine [114]. Lupine sensitization rates reported in Europe range between 0.27% and 4.1%, but these may not reflect the true prevalence of lupine allergy, which remains unknown in the general population. In a study involving 14 food allergy sources, allergic reactions were observed in 5% of the population, including 25 individuals with lupine allergy. These reactions were triggered by both discrete and cumulative doses. However, it is important to note the significant variation in the doses that caused these reactions, as indicated by the wide ranges of the confidence intervals [218]. Due to the significant variability among patients, efforts to identify the threshold doses of lupine that trigger allergic reactions have been unsuccessful [215]. SPT can be performed with the flour of all three species whereas only the *L. albus* extract is available for in vitro testing. The value of new single lupine allergens, however, has been confirmed in an optimized BAT [219] using whole blood from a patient with LTP-syndrome [220], indicating the necessity of introducing more single allergens for reliable antibody- and cell-based allergy diagnostic tests in the future.

### The “Beyond” (Cowpea, Pigeon Pea and Fenugreek)

In contrast to the legumes mentioned above, reports for cowpea, pigeon pea, and fenugreek are few, but at least some single allergens were identified in contrast to no identified allergens from Lima bean, Hyacinth bean, white pea, Guar bean, Yam-bean or Runner Bean (Table 1).

The cowpea (*Vigna unguiculata*) is an annual herbaceous plant known for its drought resilience. A study conducted in Luxemburg showed that a majority of legume allergy patients was sensitized to cowpea proteins [160]. It described four allergens with potential cross-reactivity to other legumes by in silico analysis. A previous study identified a potential allergen from the 2S albumin family in cowpea (25 kDa) using crystallographic characterization [221]. As the 2S albumin family is known for its resistance to digestion and its interaction with membranes—both crucial factors for food allergenicity—a further investigation into cowpea 2S albumins may be recommended.

The pigeon pea (*Cajanus cajan*) is a perennial legume. Despite its low intake in Europe, there has been a recent increase in its popularity due to its low cost and the growing vegan trend. The allergenicity of pigeon pea has not been extensively studied compared to peanuts and soybeans, although potential allergens from pigeon pea have already been identified by in silico analysis in 2010 without any follow-up studies [52].

Fenugreek (*Trigonella foenum-graecum*) is a legume commonly used in spice mixes as its flavor is similar to maple as well as in traditional medicine. While it was generally deemed safe for consumption, the probably first case of fenugreek allergy was reported in 1993 with occupational asthma due to inhalation of fenugreek seed powder [222] followed by reports on asthma and anaphylaxis after inhalation and skin contact [223]. In addition, oral provocation of these patients revealed its potency as food allergen source which was later confirmed [224, 225]. Two publications describe anaphylaxis to fenugreek in curry spice [226]. Four potential fenugreek allergens have been described since 2009 without further characterization [152, 227]. There is a potential cross-reactivity between fenugreek and other legumes. In 2009, Faeste and co-authors published a systematic study on a total of 31 patients, 29 with IgE to peanut, on allergenicity and possible cross-reactivity between fenugreek and other legumes. They documented an elicitation dose for fenugreek of 2 mg for the occurrence of objective allergic symptoms. They interpreted the sensitization to fenugreek as a result of peanut sensitization. Only one case of primary sensitization to fenugreek was found to date [227]. A recent study on the extent of cross-reactivity among legumes in 195 peanut-allergic children showed a sensitization to lupine, fenugreek, soy and lentils in descending order of frequency [169]. For routine diagnostics, only total fenugreek extract can currently be used in ImmunoCAP. There are no approved solutions for skin tests, i.e. only the prick-to-prick test with sponged fenugreek seed is available for detecting sensitization.

## Legume Allergy Diagnosis

Many diagnostic methods are used when investigating legume allergies, like in vivo tests (SPT, oral food challenge), functional tests in vitro and ex vivo (BAT, mast cell activation test (MAT), stripped basophil histamine release assay (BHRA), histamine release assay (HR) and lymphoproliferation assay (LPA)), in vitro non-functional tests (specific IgE, total IgE, IgE-immunoblotting, cross-reactive carbohydrate determinant (CCD)-IgE) and in silico (BLAST). Despite the increasing accuracy of both BAT and MAT most legume allergy studies have used in vitro IgE-binding assays while investigating allergens. This is due to the low number of allergic individuals. However, the recent increase of incidences might help identifying the allergenic potency of each consumed legume. It is important to provide more studies that investigate legume allergens’ stability. It is known that the sensitizing capacity and elicitation of an allergic immune response are what distinguishes allergens from non-allergenic proteins [228, 229]. However, allergens must have unique molecular properties that allow them to pass through barriers such as mucosa and skin [6]. Naturally, food processing can change the molecular structure of proteins and influence the biological activities of the respective protein [230, 231], and sometimes modify immunogenic reactivity [232]. This might increase or decrease the allergenic potential of allergens, depending on the applied process, protein structure [233–235] and whether the patient reacts towards linear and/or conformational epitopes. Physical, chemical and enzymatic processing methods have different effects on the allergenic potency of legumes [236]. However, the route of exposure via inhalation, skin (touch) or gastrointestinal tract by consumption of food, can also influence the allergenic potency. Therefore, it is important to combine knowledge about protein stability to food processing and exposure routes to be able to provide the appropriate testing method for each legume.

Introducing new products containing legume ingredients may pose risks for sensitized individuals. Furthermore, the processing methods used to produce the final product can influence the allergenicity of foods. Identifying the possible allergens in each consumed legume, and understanding their stability and the modifications they undergo (e.g. Maillard reaction) throughout the production process until the final product is obtained, will provide us with the necessary information to adjust the processing steps to an optimal level that results in a lower allergenicity of the product. This necessitates more rigorous research as the trend of vegan food is still on the rise.

## Conclusions

In addition to priority legumes (peanut, soybean), non-priority legumes (lupines, chickpea, lentils, beans) are also associated with anaphylaxis, often enough in patients allergic to priority legumes. A number of single allergens has been identified and more or less characterized from different legume families and even different legume species such as *Lupinus*. However, this knowledge on both extracts and single allergens has not been used to adapt commercially available diagnostic tests. This might become a problem in the near future as more and more individuals take to vegetarian and vegan dietary practices, therefore becoming more and more exposed to these potentially anaphylactic allergen sources. Not only are sensitization tests depending on more single allergens for improved accuracy in the detection of individual sensitization profiles, they are also important for elucidating potential cross-reactivities. In addition, as already shown for peanut and alpha-Gal, the application of single marker allergens in cell-based assays like BAT may even allow discrimination between sensitization and true allergy, providing allergologists with adequate information for individual doctoral advice for individual allergy prevention (precision medicine).
